# Effects of *Bifidobacterium animalis* subsp. *lactis* IMAU12267 on milk fermentation, subsequent storage, and functional properties

**DOI:** 10.1016/j.fochx.2026.103791

**Published:** 2026-03-26

**Authors:** Xiao Liu, Sheng Zhang, Lu Li, Yongfu Chen, Jianli Li

**Affiliations:** a Key Laboratory of Dairy Biotechnology and Engineering, Ministry of Education, Inner Mongolia Agricultural University, Hohhot, Inner Mongolia 010018, China; b Key Laboratory of Dairy Products Processing, Ministry of Agriculture and Rural Affairs, Hohhot, Inner Mongolia 010018, China; c Inner Mongolia Key Laboratory of Dairy Biotechnology and Engineering, Inner Mongolia Agricultural University, Hohhot, Inner Mongolia 010018, China

**Keywords:** *Bifidobacterium animalis* subsp. *lactis*, Fermented milk, Untargeted metabolomics, Storage stability

## Abstract

This study investigated the effects of co-fermentation with *Bifidobacterium animalis* subsp. *lactis* IMAU12267 and the commercial starter culture PYS-010 on the quality characteristics of fermented milk. Comparative evaluation of micro-rheological properties, storage stability, and untargeted metabolomic profiles among the co-fermentation (I + P), IMAU12267 (I), and PYS-010 (P) groups demonstrated that the I + P group significantly shortened fermentation time and enhanced gelation performance of the final product. The I + P group also showed higher viable counts of IMAU12267, reduced post-acidification, and superior texture, viscosity, water-holding capacity, and sensory attributes. By day 7, 14 key metabolites were identified in the I + P group, primarily linked to galactose and riboflavin metabolism and to glycolysis/gluconeogenesis pathways, indicating improvements in flavor, product quality, and nutritional value. These results reveal a synergistic metabolic interaction between IMAU12267 and PYS-010 and offer valuable insight for the development of high-quality, functionally enhanced fermented dairy products.

## Highlights

• IMAU12267 increases α-lactose and D-galactose to improve fermented milk quality.
• IMAU12267 elevates riboflavin and capric acid to enhance nutritional functionality.
• IMAU12267 lowers acetic acid levels to support balanced flavor profiles.

## 1 Introduction

Fermented milk is among the oldest and most distinctive dairy foods, produced through the activity of lactic acid bacteria (LAB) that convert lactose into lactic acid. As fermentation progresses, the pH declines to the isoelectric point of casein, initiating casein aggregation and gel network formation that ultimately results in milk coagulation (Wu et al., 2023). Compared with fresh milk, fermented milk offers a unique flavor profile, a desirable texture, an improved mouthfeel, enhanced nutritional properties, and diverse health benefits. Reported advantages include modulation of gut microbiota composition, mitigation of lactose intolerance, enhancement of immune responses, and reductions in serum cholesterol concentrations. Owing to these attributes, fermented milk has gained widespread global acceptance (Yang et al., 2008). Industrial production is largely dependent on commercial starter cultures, typically composed of *Streptococcus thermophilus* and *Lactobacillus delbrueckii* subsp. *bulgaricus*. However, as consumer demand shifts toward functional foods and more complex flavor experiences, conventional fermented milk products with relatively simple sensory characteristics have increasingly struggled to satisfy evolving market expectations (Zha et al., 2021). Therefore, incorporating functional LAB strains into fermented milk has become an important strategy for developing innovative, high-quality, and health-promoting dairy products.

Among these functional microorganisms, bifidobacteria constitute a major group of beneficial LAB naturally inhabiting the mammalian gastrointestinal tract. They contribute to intestinal barrier maintenance, inhibit pathogen adhesion, and synthesize essential metabolites and vitamins that support host health (Zhong et al., 2025). Bifidobacteria typically colonize the gut early in life, adhering closely to the intestinal mucosa to establish stable microbial communities. In healthy breastfed infants, *Bifidobacterium* species may account for approximately 60–70% of total gut microbiota, whereas their abundance decreases with age, averaging about 10% in adults and roughly 5% in elderly populations (Xiao et al., 2024). Therefore, enhancing dietary intake of *Bifidobacterium* through food-based delivery systems is of considerable importance, and fermented milk represents an effective carrier matrix for these beneficial microorganisms (Rodriguez-Minguez et al., 2024). Increasing evidence highlights the physiological benefits of *Bifidobacterium animalis* subsp. *lactis* in fermented milk products. For example, Yang et al. (2025) reported that oral administration of fermented milk containing *B. animalis* subsp. *lactis* BX-245 significantly altered gut and tumor-associated microbiota, modulated metabolic pathways, and reduced tumor burden in a murine colorectal cancer model. Guyonnet et al. (2007) demonstrated that fermented milk enriched with *B. animalis* DN-173010 alleviated bloating, abdominal pain, and digestive discomfort in individuals with irritable bowel syndrome. Freitas et al. (2024) further showed that fermented milk supplemented with *B. animalis* subsp. *lactis* BB-12 reduced anxiety-like behavior and improved sleep quality in Wistar–Kyoto rats. These findings emphasize the substantial potential of *B. animalis* subsp. *lactis* to promote health through fermented dairy consumption. However, despite these advantages, industrial utilization of bifidobacteria in dairy fermentation remains constrained by their relatively slow growth in milk substrates and their strict requirement for anaerobic, low-redox conditions, factors that prolong fermentation time and reduce process efficiency (Prasanna et al., 2014). Co-fermentation with conventional starter cultures has therefore been proposed as a practical approach to overcome these limitations.

Within co-fermentation systems, commercial starter strains can rapidly establish a favorable microenvironment that enhances bifidobacterial metabolic activity and helps maintain their functional predominance during fermentation. In this context, a systematic investigation of compositional and dynamic metabolite changes in co-fermented milk during storage can provide valuable molecular-level insight into bifidobacterial functionality and the mechanisms underlying product quality in these products. In recent years, untargeted metabolomics has emerged as a powerful analytical strategy for characterizing microbial metabolism in fermented milk (Xia et al., 2020). For instance, Mi et al. (2018) profiled metabolic shifts during milk fermentation by *Lactobacillus helveticus* H9 and identified key metabolites, including amino acids, peptides, vitamins, and organic acids. Similarly, Wang et al. (2024) compared camel milk with fermented camel milk and reported significantly elevated guanine concentrations in the fermented samples. Similarly, Jingjing et al. (2025) investigated flavor evolution in pasteurized yogurt during storage. They found that metabolic transformations were primarily associated with starch and sucrose metabolism, amino sugar and nucleotide sugar metabolism, and purine metabolism pathways. These findings demonstrate that untargeted metabolomics can effectively elucidate the complex biochemical processes occurring in fermented milk during storage. However, most previous studies of bifidobacterial co-fermentation have focused mainly on conventional physicochemical and microbiological indicators. At the same time, detailed kinetic analyses of gel formation and mechanistic links between storage stability and metabolite dynamics remain limited. Furthermore, bifidobacterial performance in milk matrices is often strain dependent (Prasanna et al., 2014), underscoring the importance of metabolite-level evidence to clarify the behavior of individual strains in co-fermentation systems.

Based on this background, the present work examined a co-fermentation system composed of *B. animalis* subsp. *lactis* IMAU12267 (I) and the commercial starter culture PYS-010 (P), comparing their performance with fermentations conducted using IMAU12267 or PYS-010 alone to systematically evaluate the influence of co-fermentation on fermentation characteristics and storage quality. The IMAU12267 strain employed in this study was isolated in 2022 from fecal samples of healthy infants in Hohhot, Inner Mongolia, whereas PYS-010 is widely applied as a commercial starter in industrial dairy processing. Microrheological analysis was first used to characterize gelation behavior and quantify the effects of co-fermentation on gel network formation and fermentation kinetics. Afterwards, pH, titratable acidity (TA), viable cell counts, viscosity, water-holding capacity (WHC), texture, and sensory attributes were monitored throughout storage to evaluate post-acidification and overall quality stability. Finally, to elucidate the molecular mechanisms underlying storage-related quality differences, untargeted metabolomic profiling was performed on samples from the three groups collected on day 7 of storage. This study aims to provide a theoretical basis for developing novel functional fermented milk products enriched with *B. animalis* subsp. *lactis.*

## 2 Materials and methods

### 2.1 Experimental strains

*Bifidobacterium animalis* subsp. *lactis* IMAU12267 was obtained from the Lactic Acid Bacteria Culture Collection of Inner Mongolia Agricultural University. The strain was originally isolated in 2022 from fecal samples of healthy infants in Hohhot, Inner Mongolia Autonomous Region. The commercial starter culture PYS-010, which contains *Streptococcus salivarius* subsp. *thermophilus* and *Lactobacillus delbrueckii* subsp. *bulgaricus* was purchased from Beijing Scitop Biotechnology Co., Ltd., Beijing.

### 2.2 Fermented milk production

Commercial milk supplied by Inner Mongolia Mengniu Dairy Co., Ltd. (Hohhot, China), which accounted for 93.5% of the total formulation, was preheated to 65 °C. White granulated sugar from Guangxi Fengtang Biochemical Co., Ltd. (Liuzhou, China), representing 6.5% of the total volume, was then added and stirred continuously for 15 min until fully dissolved. The mixture was homogenized at 20 MPa, pasteurized at 95 °C for 5 min, and then rapidly cooled to the inoculation temperature. The designated starter cultures were subsequently inoculated and thoroughly blended, after which fermentation was carried out at 37 °C until the pH reached 4.5 ± 0.1. At the end of fermentation, samples were promptly cooled, stirred, packaged, and stored at 4 °C for 21 days. Three experimental groups were prepared: the co-fermentation group (I + P), inoculated with *B. animalis* subsp. lactis IMAU12267 (5.0 × 10^6^ CFU/mL) and PYS-010 (0.003%, *w*/*v*); the I group, containing only IMAU12267 (5.0 × 10^6^ CFU/mL); and the P group, containing only PYS-010 (0.003%, w/v).

### 2.3 Evaluation of micro-rheological properties

The fermentation process was continuously monitored using a micro-rheometer (Rheolaser Master, Beijing, China). A 20 mL portion of inoculated milk was transferred into a sterile sample vial (inner diameter: 27.5 mm) and placed in the instrument's sample chamber. Measurements were performed at 37 °C. Temporal variations in the Elasticity Index (EI), Fluidity Index (FI), Solid–Liquid Balance (SLB), and Macroscopic Viscosity Index (MVI) were recorded at 5 min intervals throughout fermentation. Data acquisition ceased once fermentation was complete, and the resulting micro-rheological parameters were analyzed using the instrument's dedicated software.

### 2.4 Storage stability of fermented milk

#### 2.4.1 Determination of viable counts

The viable population of *B. animalis* subsp. *lactis* IMAU12267 in fermented milk samples was quantified following the procedure described by Zhong et al. (2025). Briefly, 10 g of the sample was homogenized with 90 mL of sterile physiological saline and agitated for 15 min at 4 °C to achieve uniform dispersion. Serial dilutions were subsequently prepared using sterile saline. An aliquot (1 mL) of an appropriate dilution was transferred to a sterile Petri dish, and 15 mL of ML agar was added. The mixture was gently swirled, allowed to solidify, and incubated anaerobically at 37 °C for 48 h. Thereafter, the resulting colonies were enumerated to determine the viable cell count.

#### 2.4.2 Assessment of pH and TA

The pH and titratable acidity (TA) of fermented milk samples were determined following the method described by Bai et al. (2024). The pH was measured directly using an SJ-3F pH meter (Shanghai, China). For TA analysis, 10 g of sample was combined with 20 mL of distilled water and 2 mL of phenolphthalein indicator, mixed thoroughly, and titrated with standardized 0.1 mol/L NaOH until a faint pink endpoint appeared and persisted for 30 s. The volume of NaOH consumed was then used to calculate titratable acidity (°T) according to formula (1).

(1) $$\mathrm{TA}\;\left({}^{{}^{\circ}}T\right)=\left(V\times 100\right)/m$$

V: The volume of NaOH solution used, computed in milliliters (mL).
M: The mass of the sample, calculated in grams (g).

#### 2.4.3 Assessment of water-holding capacity and viscosity

The viscosity and water-holding capacity (WHC) of fermented milk samples were evaluated following the method described by Bai et al. (2024). Viscosity was determined using a Brookfield DV2T viscometer (Brookfield Engineering Laboratories Inc., Middleboro, MA, USA) fitted with a No. 4 spindle operating at 100 rpm. Readings were recorded after 30 s of continuous rotation, ensuring that torque values remained within 10%–100% of the initial setting. For WHC analysis, 20 g of each sample was transferred into a funnel lined with filter paper and allowed to stand at room temperature for 2 h. The expelled whey was then collected and weighed, and WHC was calculated using eq. (2).

(2) $$\mathrm{WHC}\;\left(\%\right)=\left[1-\left(\text{filtrate mass},g/\text{sample mass},g\right)\right]\times 100\%$$

#### 2.4.4 Determination of texture characteristics

The texture characteristics of fermented milk samples were evaluated using a TA.XT Plus texture analyzer (Stable Micro Systems, UK). Before analysis, the A/BE probe was carefully cleaned to maintain measurement precision. An appropriate volume of sample was then placed into the designated sample cup, and the instrument's built-in yogurt consistency testing program was applied. All operating conditions were set according to Bai et al. (2024): trigger force, 2.0 g; compression ratio, 20%; compression time, 5 s; test distance, 20 mm; pre-test and post-test speeds, 1.5 mm/s; and test speed, 1.0 mm/s.

#### 2.4.5 Sensory assessment

The sensory assessment was conducted by a panel of 10 trained dairy professionals, all of whom had passed a sensory qualification test before participation. Fermented milk samples were thoroughly homogenized, dispensed into tasting cups according to treatment group, and presented to panelists for assessment. The evaluation was conducted using a paper-based questionnaire that encompassed four attributes: color, taste, aroma, and structural consistency (texture). Detailed scoring criteria are listed in Table 1. Although ethical approval was not required for this sensory study, all panelists were fully informed of the purpose and procedures and provided informed consent to participate. No personal identifiers were collected, and all data were recorded and analyzed anonymously to protect participants' rights and privacy.

**Table 1:** Sensory scores of fermented milk during storage.

Sensory item	Feature	Score
Color (20 points)	The color is uniform and consistent, appearing milky white or creamy yellow in hue.	12–20
	Uneven color, with deep yellow or gray.	4–11
	Uneven color, colored spots or impurities, or other abnormal colors.	0–3
Taste and smell (40 points)	Authentic milk flavor, with natural fermentation taste and aroma, balanced sweetness and acidity.	31–40
	The natural fermentation flavor is insufficient, slightly sour, or slightly sweet.	21–30
	Not enough milky flavor, poor natural fermentation taste, with bitterness, too sour, or too sweet.	5–20
	Unpleasant smell.	0–4
Organizational status (40 points)	The texture is delicate and even, with a smooth, flat surface free of cracks. The cut surface is also smooth and flat, demonstrating a solid texture with good elasticity. There is no powdery feel, no pasty taste, and no bubbles or whey separation.	31–40
	The surface is flat but not smooth, with slightly visible particles to the naked eye. There are no apparent cracks. The cut surface is flat but slightly rough, with a few bubbles appearing or slight whey separation.	21–30
	The texture is rough, with visible particles to the naked eye, evident cracks, occasional small curd lumps on the surface, uneven cut surface, soft texture, poor elasticity, a pasty mouthfeel, and visible bubbles or significant whey separation.	5–20
	Rough texture, severe visible particles, numerous cracks, curd pieces of varying sizes, no clear-cut surface, soft texture, no elasticity, strong pasty mouthfeel, and numerous bubbles or severe whey separation.	0–4

Remarks: The taste and aroma do not involve sweetness; only the sourness is evaluated.

### 2.5 Untargeted metabolomics analysis of fermented milk

#### 2.5.1 Sample pretreatment

Fermented milk samples stored at −80 °C were thawed at 4 °C before analysis. Each 3 mL sample was mixed with 1 mL of acetonitrile and vortexed thoroughly. The mixture was then centrifuged at 10,000*g* for 10 min at 4 °C. Subsequently, 1 mL of the supernatant was combined with 3 mL of acetonitrile, vortexed again, and maintained at 4 °C for 2 h. Afterward, the solution was centrifuged at 12,000*g* for 5 min, and the resulting supernatant was collected and concentrated by rotary evaporation for 9 h. The dried residue was reconstituted in 400 μL of 40% (*v*/v) acetonitrile–water solution, filtered through a 0.22 μm membrane, and subjected to subsequent analysis. For quality control purposes, equal aliquots from each sample were pooled to generate QC samples. Before sample measurement, acetonitrile was analyzed as a blank, and both blank and QC samples were included to evaluate chromatographic stability and analytical reproducibility (Begou et al., 2018).

#### 2.5.2 LC-MS chromatographic conditions

Metabolite profiling was performed using a UPLC–Q-TOF-MS 6600+ platform. Chromatographic separation was carried out on an Acquity UPLC® HSS T3 column (1.8 μm, 2.1 mm × 100 mm), maintained at 35 °C, with a flow rate of 0.35 mL/min and an injection volume of 2.0 μL. Under positive electrospray ionization (ESI^+^) conditions, mobile phase A comprised ultrapure water with 0.1% formic acid, and mobile phase B consisted of acetonitrile containing 0.1% formic acid. In negative ion mode (ESI^−^), mobile phase A was ultrapure water with 0.1% ammonia, while mobile phase B was pure acetonitrile. The gradient program (total run time 23 min) was defined as follows: 0–0.25 min, 5% B; 0.25–1 min, 5% B; 1–6 min, 5–40% B; 6–18 min, 40–85% B; 18–20 min, 85–90% B; 20–22 min, 90% B; 22–22.5 min, 5% B; and 22.5–23 min, 5% B. Mass spectrometric detection was conducted in both ESI^+^ and ESI^−^ modes. The ion source temperature was set to 550 °C, with source voltages of +5500 V for ESI^+^ and − 4500 V for ESI^−^. Gas parameters were configured as follows: ion source gas I, 50 psi; ion source gas II, 35 psi; and curtain gas, 25 psi. The declustering potential was ±60 V for both ionization modes, and the collision energy was maintained at 35 eV.

#### 2.5.3 Metabolomics data processing

Raw mass spectrometry data were processed using Progenesis QI 2.2 software, incorporating baseline correction, peak alignment, feature detection and extraction, deconvolution, and normalization. Putative metabolite identification was achieved by comparing processed features with multiple reference databases, including HMDB, KEGG, and ChemSpider. The resulting metabolite dataset was then uploaded to the MetaboAnalyst 5.0 online platform for multivariate statistical analysis. Principal component analysis (PCA) and partial least squares discriminant analysis (PLS-DA) were applied to identify and discriminate differential metabolites.

### 2.6 Multivariate statistical analysis

All experiments were performed in triplicate. Data were compiled using Microsoft Excel 2019, and statistical analyses and graphical presentations were generated with Origin 2023. Group differences were evaluated using a one-way analysis of variance (ANOVA) in IBM SPSS Statistics 23.0, with *P* < 0.05 considered significant.

## 3 Results

### 3.1 Micro-rheological properties of fermented milk

**Fig. 1:**
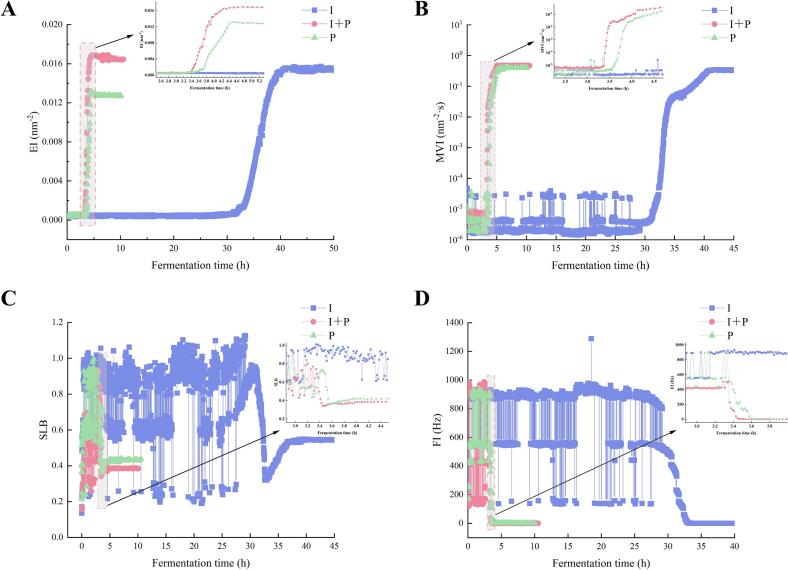
Changes in the micro-rheological properties of fermented milk: (A) Elasticity Index, (B) Macroscopic Viscosity Index, (C) Solid-Liquid Balance, and (D) Fluidity Index.

Assessment of micro-rheological parameters provides an effective approach for tracking the liquid-to-gel transition of the milk matrix throughout fermentation. In this study, four principal indicators, EI, MVI, SLB, and FI, were analyzed (Fig. 1). The Elasticity Index (EI) describes time-dependent changes in sample elasticity, with higher values indicating stronger structural resilience and stability. The MVI reflects apparent viscosity at low shear, and elevated MVI values indicate increased viscosity. The SLB indicates the relative dominance of solid-like versus liquid-like behavior within the system: a value of 0 represents a purely elastic solid; 0–0.5 denotes predominantly solid characteristics; 0.5 corresponds to the critical liquid–solid transition point; 0.5–1 reflects partial liquid behavior; and ≥ 1 indicates a fully liquid state. Accordingly, lower SLB values signify reduced particle mobility and a more solid-like network structure. The FI characterizes flow properties, with lower values reflecting decreased fluidity and weaker flow behavior *.*

At the initial stage of fermentation, EI and MVI values were low in all groups. At the same time, SLB and FI remained high, with only slight variation, indicating that the systems predominantly exhibited liquid-like behavior. As fermentation progressed, the I + P group showed substantially improved gelation performance compared with the I group. This enhancement is likely associated with *B. animalis* subsp. *lactis* IMAU12267, which may stimulate exopolysaccharide (EPS) synthesis, strengthening micro-rheological stability and promoting gel network development in the co-fermentation system. The SLB of the I + P group decreased below 0.5 at approximately 3.4 h, indicating an earlier gelation onset than observed in the P and I groups, which reached this transition at about 3.6 h and 32.6 h, respectively (Fig. 1C). Similarly, EI and MVI values in the I + P group rose sharply (Fig. 1A, B). In comparison, FI declined rapidly and stabilized near 0.01 (Fig. 1D). In comparison, the P and I groups showed lower peak EI and MVI values, more gradual reductions in SLB, and delayed stabilization of FI. These findings indicate that incorporation of IMAU12267 into the co-fermentation system promotes gel network formation and improves micro-rheological properties, yielding fermented milk with enhanced elasticity, viscosity, and structural integrity.

### 3.2 Storage stability of fermented milk

#### 3.2.1 Determination of fermentation time and viable cell counts of fermented milks

**Fig. 2:**
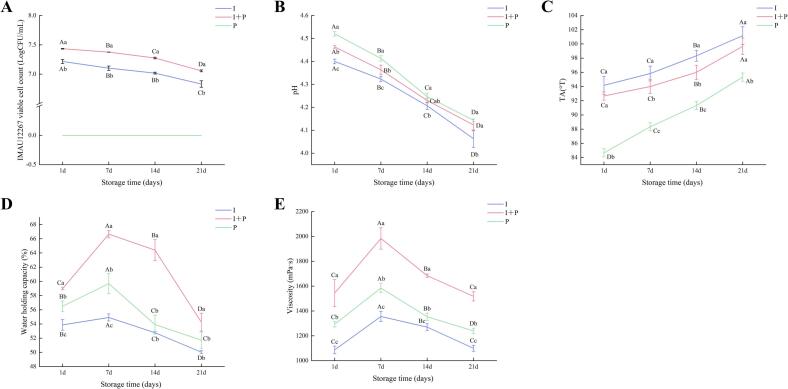
Properties of fermented milk made with commercial starter PYS-010 (P), IMAU12267 (I), or their combination (I + P). Changes in (A) viable counts of IMAU12267, (B) pH value, (C) titratable acidity, (D) water-holding capacity, and (E) viscosity. Different uppercase letters indicate significant differences within the same group, while different lowercase letters indicate significant differences between groups (*P* < 0.05). Error bars represent the standard deviation.

Fermentation time and viable cell counts are critical indicators for evaluating both manufacturing efficiency and storage stability of fermented milk. Fermentation time directly influences industrial productivity and processing costs, as prolonged fermentation increases energy demand and decreases operational efficiency. At the same time, the viable cell counts of lactic acid bacteria serves as a key determinant of product quality and stability during storage. In this study, both fermentation time and bacterial viability were systematically assessed. Fermentation time decreased significantly from 25.52 h in the I group and 6.04 h in the P group to 5.50 h in the I + P group (*P* < 0.05) (Fig. S1A), demonstrating a significant enhancement in fermentation efficiency. Throughout storage, viable cell counts gradually declined in both the I and I + P groups; however, by day 21, the I + P group retained a significantly higher population of *B. animalis* subsp. *lactis* IMAU12267 (1.14 × 10^7^ CFU/mL) than the I group (6.79 × 10^6^ CFU/mL, *P* < 0.05) (Fig. 2A). This outcome suggests that co-fermentation with PYS-010 may establish a more favorable microenvironment for IMAU12267, possibly by lowering dissolved oxygen levels, reducing oxidative stress, and improving bacterial survival during storage. Therefore, co-fermentation not only enhances processing efficiency but also promotes microbial viability and overall product stability. Similar protective effects of co-fermentation bifidobacteria with starter cultures have been previously reported by Odamaki et al. (2011).

#### 3.2.2 Determination of pH and TA

The acidification profile of fermented milk during storage serves as an important indicator of microbial performance and metabolic activity (Li, Lu, et al., 2025). Over the storage period, all three groups showed a progressive decrease in pH, accompanied by a corresponding increase in TA, reflecting post-acidification resulting from ongoing LAB metabolism (Fig. 2B, C). Among the groups, the I group showed the most pronounced post-acidification, with pH declining to 4.06 and TA reaching its highest level by day 21. Excessive acidification can negatively affect product quality by diminishing flavor smoothness and weakening gel stability. In comparison, the I + P group displayed a more controlled acidification pattern, evidenced by a smaller pH decline to 4.12 after 21 days and a more gradual rise in TA. These findings suggest that co-fermentation effectively attenuated the excessive post-acidification observed in the I group, indicating a synergistic metabolic interaction between IMAU12267 and PYS-010. All fermented milk samples maintained TA values within the consumer-acceptable range (70–110°T) at the end of storage, confirming that all products retained desirable acidity levels and overall quality. These results demonstrate that co-fermentation with PYS-010 not only regulates the acidification kinetics of IMAU12267 but also supports improved flavor stability and product uniformity during refrigerated storage.

#### 3.2.3 Determination of WHC and viscosity

The WHC and viscosity are key physicochemical attributes that influence the quality, texture, structural integrity, and consumer acceptance of fermented milk. WHC represents the product's capacity to retain moisture, whereas viscosity determines thickness and mouthfeel. As illustrated in Fig. 2D and E, both parameters increased during the early storage phase (days 1–7) and then gradually declined between days 7 and 21 in all groups. Across the entire storage period, the I + P group consistently demonstrated significantly higher WHC and viscosity than the I and P groups (*P* < 0.05). Peak values were observed on day 7, when WHC reached 66.65%, and viscosity attained 1984 mPa·s. These findings suggest that the synergistic interaction between *B. animalis* subsp. *lactis* IMAU12267 and PYS-010 enhance moisture retention and viscosity, improving texture and overall structural quality in the fermented milk product.

#### 3.2.4 Determination of texture characteristics

**Fig. 3:**
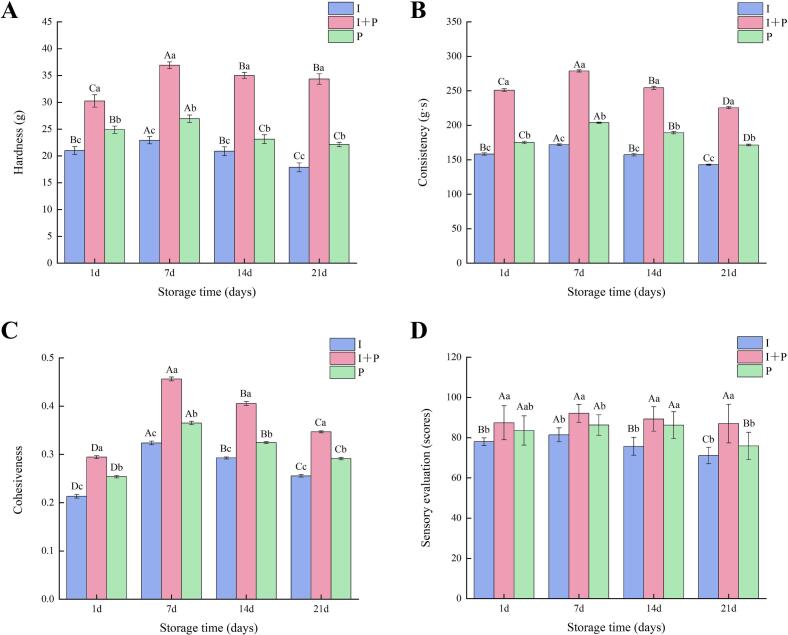
Textural characteristics and sensory scores of fermented milk prepared using commercially available starter PYS-010 (P), IMAU12267 (I), or their combination (I + P). Changes in (A) Hardness, (B) Consistency, (C) Cohesiveness, (D) Sensory scores. Different uppercase letters indicate significant differences within the same group, while different lowercase letters indicate significant differences between groups (*P* < 0.05). Error bars represent the standard deviation.

Hardness, consistency, and cohesiveness are key textural indicators for assessing the structural integrity and stability of fermented milk. In three groups, these parameters increased during the early storage phase and then gradually declined over time. This trend corresponded closely with the patterns observed for WHC and viscosity (Fig. 3A–C), suggesting that significant quality changes emerged around day 7 of storage. At this stage, the I + P group showed peak textural values, with hardness, consistency, and cohesiveness reaching 36.91 g, 278.73 g·s, and 0.46, respectively, all significantly higher than those recorded for the I and P groups (*P* < 0.05). During the later storage period, intensified post-acidification likely induced ionic rearrangements within the protein network, weakening the gel matrix and leading to reductions in hardness, consistency, and cohesiveness. However, by the end of storage, the I + P group maintained the highest textural values, indicating superior structural stability of the gel network. These findings suggest that synergistic interactions between IMAU12267 and PYS-010 facilitate the formation of a stronger, more stable gel structure, reducing whey separation and mitigating texture deterioration associated with post-acidification.

#### 3.2.5 Sensory evaluation

Sensory evaluation serves as an essential link between the physicochemical attributes of fermented milk and its consumer acceptance. Across the storage period, the I + P group consistently obtained significantly higher sensory scores than the I and P groups (*P* < 0.05). Scores for the I + P samples remained above 85 throughout storage, indicating a high degree of overall acceptability, and peaked at 92.1 on day 7 (Fig. 3D). At this point, the co-fermented samples revealed uniform coloration, a smooth and delicate texture, balanced acidity, and a characteristic fermented aroma, highlighting the synergistic effects of combining IMAU12267 with PYS-010. Co-fermentation of these strains significantly improved sensory quality, yielding a fermented milk product with a harmonious flavor profile, refined mouthfeel, and strong consumer appeal.

### 3.3 Untargeted metabolomics analysis of fermented milk

#### 3.3.1 PCA and PLS-DA

**Fig. 4:**
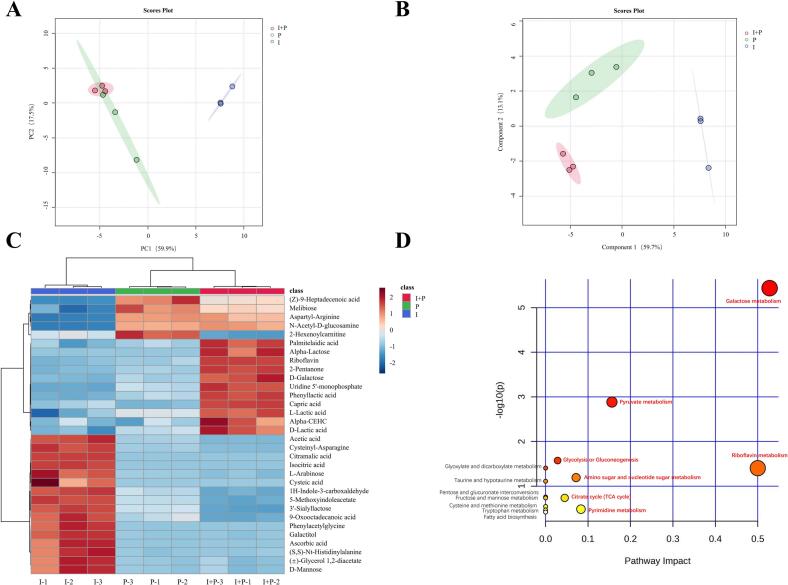
Differential metabolites and their associated pathways identified by metabolomic analysis of fermented milk after storage for seven days. Fermented milk samples were prepared using the commercial starters PYS-010 (P) and IMAU12267 (I), or their co-fermentation (I + P). (A) Principal component analysis (PCA) and (B) partial least squares discriminant analysis (PLS-DA) score plots illustrating the overall metabolic differences among the three groups. (C) Heatmap showing the relative abundance of significantly differentially expressed metabolites, with color gradients from low (blue) to high (red). (D) Bubble plot of enriched metabolic pathways, with the bubble size representing both the effect value of the pathway and the number of enriched metabolites, based on topological analysis. Larger bubbles represent pathways with greater influence, and the bubbles' hues indicate log (*P*) values, with deeper colors denoting greater statistical significance. (For interpretation of the references to color in this figure legend, the reader is referred to the web version of this article.)

Comprehensive assessment of storage quality identified day 7 as a representative time point at which intergroup differences were most clearly defined. Therefore, samples collected on day 7 were selected for untargeted metabolomic analysis. To verify data reliability, total ion chromatograms of QC samples were first examined in both ESI^+^ and ESI^−^ modes. The high degree of chromatographic overlap indicated excellent instrument stability and experimental reproducibility, confirming the reliability of the subsequent metabolomic dataset (Fig. S1B, C). Moreover, PCA showed partial separation of the three groups along PC1, with some overlap (Fig. 4A), suggesting that although the groups shared certain metabolic characteristics, distinct metabolic differences remained. To improve classification performance, partial least squares discriminant analysis (PLS-DA) was applied. PLS-DA explained 73.8% of the total variance across the first two principal components and effectively distinguished the three groups without significant outliers, indicating intrinsic metabolic differentiation among them (Fig. 4B). Model validation further demonstrated robustness, with cross-validation metrics of R^2^ = 0.99 and Q^2^ = 0.84 (Fig. S1D). Permutation testing supported these results (Fig. S1E), confirming strong explanatory and predictive power without evidence of overfitting. These results establish the suitability of the model for subsequent identification and interpretation of differential metabolites.

#### 3.3.2 Enrichment analysis of the differential metabolites and pathways

Untargeted metabolomic profiling (Section 3.3.1) identified 32 metabolites that differed significantly among the three fermented milk groups on day 7 of storage (FDR < 0.05, VIP > 1; Fig. 4C). These compounds comprised nine organic oxygen compounds, nine organic acids and derivatives, seven lipids and lipid-like molecules, five organoheterocyclic compounds, one benzenoid compound, and one nucleotide or nucleotide analogue (Table 2). Their relative abundances varied significantly across the groups. Pathway enrichment analysis indicated that these differential metabolites were primarily involved in 14 major metabolic pathways (Fig. 4D), including galactose metabolism, pyruvate metabolism, riboflavin metabolism, glycolysis/gluconeogenesis, amino sugar and nucleotide sugar metabolism, the citrate (TCA) cycle, pyrimidine metabolism, glyoxylate and dicarboxylate metabolism, taurine and hypotaurine metabolism, pentose and glucuronate interconversions, fructose and mannose metabolism, cysteine and methionine metabolism, tryptophan metabolism, and fatty acid biosynthesis.

**Table 2:** Differential metabolite identification of three groups of fermented milk samples.

Biochemical Classes	Metabolites	Molecular formula	Mass-to-Charge Ratio (*m*/*z*)	Retention Time (min)	False Discovery Rate	VIP score
Organic oxygen compounds	Melibiose	C_12_H_22_O_11_	341.11	0.96	4.37E−03	16.19
	D-Mannose	C_6_H_12_O_6_	161.05	0.92	2.47E−05	6.57
	2-Pentanone	C_5_H_10_O	131.07	4.65	1.20E−02	3.29
	Alpha-Lactose	C_12_H_22_O_11_	341.11	4.12	1.46E−02	1.48
	N-Acetyl-D-glucosamine	C_8_H_15_NO_6_	220.08	1.00	3.13E−02	1.56
	Galactitol	C_6_H_14_O_6_	181.07	0.93	1.68E−02	1.25
	D-Galactose	C_6_H_12_O_6_	215.03	0.88	3.25E−03	1.83
	3’-Sialyllactose	C_23_H_39_NO_19_	632.20	0.86	1.40E−05	4.88
	L-Arabinose	C_5_H_10_O_5_	195.05	0.84	2.68E−04	1.45
Organic acids and derivatives	Isocitric acid	C_6_H_8_O_7_	191.02	0.81	3.23E−05	13.28
	D-Lactic acid	C_3_H_6_O_3_	89.02	0.90	1.05E−02	6.31
	Phenylacetylglycine	C_10_H_11_NO_3_	214.05	0.90	1.03E−05	2.61
	Acetic acid	C_2_H_4_O_2_	179.06	0.93	1.38E−03	2.26
	Cysteic acid	C_3_H_7_NO_5_S	189.98	1.32	5.71E−03	2.17
	Aspartyl-Arginine	C_10_H_19_N_5_O_5_	310.11	0.97	1.19E−05	5.99
	L-Lactic acid	C_3_H_6_O_3_	269.09	0.92	4.64E−02	1.70
	(*S*, *S*)-Nt-Histidinylalanine	C_9_H_14_N_4_O_4_	263.08	0.92	8.27E−05	3.60
	Cysteinyl-Asparagine	C_7_H_13_N_3_O_4_S	515.12	0.81	1.23E−03	1.72
Lipids and lipid-like molecules	Citramalic acid	C_5_H_8_O_5_	147.03	0.81	2.98E−05	4.30
	(±)-Glycerol 1,2-diacetate	C_7_H_12_O_5_	157.05	4.87	7.97E−03	1.88
	Palmitelaidic acid	C_16_H_30_O_2_	253.22	20.13	2.18E−02	1.41
	(Z)-9-Heptadecenoic acid	C_17_H_32_O_2_	267.23	21.02	1.52E−04	1.05
	9-Oxooctadecanoic acid	C_18_H_34_O_3_	297.24	16.21	3.20E−02	1.41
	Capric acid	C_10_H_20_O_2_	362.33	11.07	2.33E−02	2.30
	2-Hexenoylcarnitine	C_13_H_23_NO_4_	256.16	8.721	1.89E−02	1.14
Organoheterocyclic compounds	Ascorbic acid	C_6_H_8_O_6_	175.02	0.81	1.74E−04	2.41
	5-Methoxyindoleacetate	C_11_H_11_NO_3_	204.07	5.40	1.55E−02	2.60
	1H-Indole-3-carboxaldehyde	C_9_H_7_NO	144.05	7.35	1.06E−02	1.47
	Alpha-CEHC	C_16_H_22_O_4_	301.14	15.44	5.40E−03	1.05
	Riboflavin	C_17_H_20_N_4_O_6_	377.14	5.12	2.00E−02	1.16
Benzenoids	Phenyllactic acid	C_9_H_10_O_3_	147.04	4.99	6.76E−03	1.40
Nucleosides, nucleotides, and analogues	Uridine 5′-monophosphate	C_9_H_13_N_2_O_9_P	305.02	0.88	4.05E−02	2.20

Figure captions.

#### 3.3.3 Differential metabolites specific to the I + P group

**Fig. 5:**
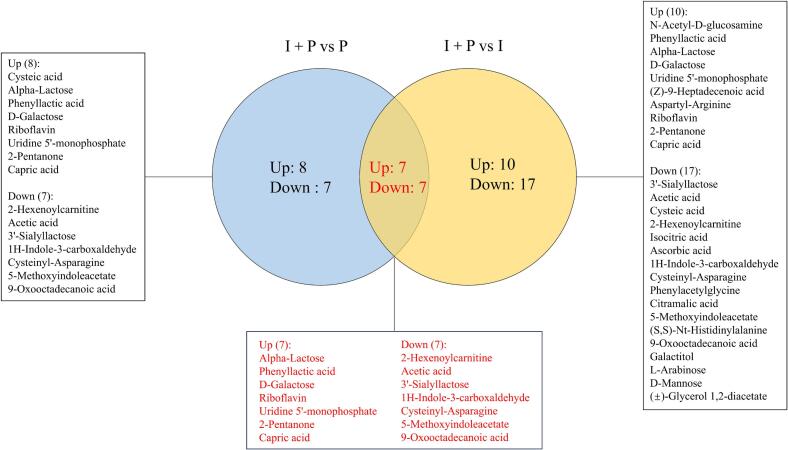
The Venn diagram showing the unique and common differential metabolites in fermented milk at 7 days of storage.

**Fig. 6:**
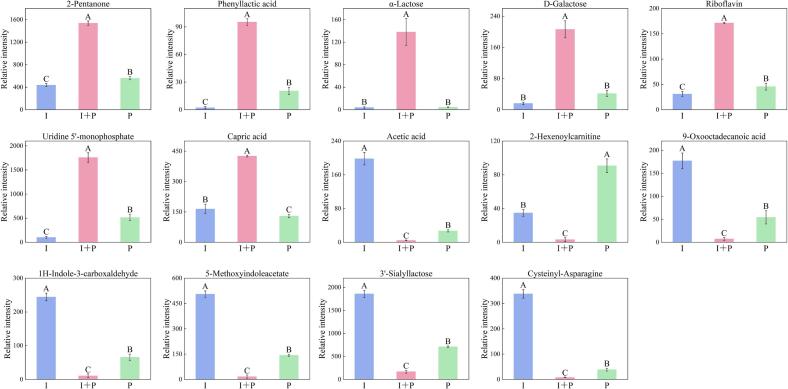
Fermented milk samples prepared using the commercial starter PYS-010 (P), IMAU12267 (I), or their co-fermentation (I + P). Different uppercase letters indicate significant differences within the same group (*P* < 0.05). Error bars represent the standard deviation.

To further elucidate the synergistic metabolic interactions underlying co-fermentation by IMAU12267 and PYS-010, fold-change (FC) analysis (FC > 2 or FC < 0.5) was applied to the 32 differential metabolites. Pairwise comparisons of I + P versus I and I + P versus P revealed distinct metabolic alterations. Relative to the P group, the I + P group showed increased levels of eight metabolites and decreased levels of seven. Similarly, compared with the I group, the I + P group showed elevated intensities for 10 metabolites and reduced intensities for 17. A Venn diagram analysis was subsequently performed to identify shared differential metabolites, yielding 14 core compounds characteristic of the I + P group (Fig. 5). Among these, seven metabolites, α-lactose, phenyllactic acid, D-galactose, riboflavin, uridine 5′-monophosphate, 2-pentanone, and capric acid, displayed consistently higher relative abundances. In comparison, seven others, 2-hexenoylcarnitine, acetic acid, 3′-sialyllactose, 1H-indole-3-carboxaldehyde, cysteinyl-asparagine, 5-methoxyindoleacetate, and 9-oxooctadecanoic acid, showed reduced levels (Fig. 6). These 14 metabolites define the distinctive metabolic signature of the I + P co-fermentation system. Their coordinated abundance shifts provide compelling molecular evidence of synergistic metabolic regulation between IMAU12267 and PYS-010, clarifying the biochemical basis for the enhanced quality observed in the co-fermented milk.

## 4 Discussion

*Bifidobacterium* species, among the dominant genera in the human gut microbiota, play essential roles in modulating innate and adaptive immune responses, regulating host metabolism, and influencing gut–brain axis signaling (He et al., 2023). Fermented milk serves as an effective carrier for delivering *Bifidobacterium*, supporting its incorporation into the gastrointestinal microbial community. However, the strict nutritional demands and environmental sensitivity of *Bifidobacterium* in milk-based substrates pose significant challenges for its large-scale industrial application in fermented dairy manufacturing (Prasanna et al., 2014). For this reason, co-fermentation of bifidobacteria with starter cultures is widely considered a practical approach to enhance their adaptability and fermentation performance. Most existing research has primarily emphasized conventional physicochemical and microbiological comparisons, while comprehensive analyses integrating gelation kinetics during structure development, storage stability, and metabolite dynamics remain limited. Moreover, the growth characteristics and metabolic behavior of bifidobacteria during milk fermentation are often strain-specific (Prasanna et al., 2014), underscoring the importance of systematic evaluation using defined strains. Addressing these knowledge gaps, this study systematically investigated the effects of co-fermentation with *B. animalis* subsp. *lactis* IMAU12267 and the commercial starter PYS-010 on rheological behavior, storage stability, and untargeted metabolomic profiles of fermented milk. The findings offer important insight into the role of IMAU12267 in improving both the quality attributes and functional potential of fermented dairy products.

Micro-rheological parameters monitored during fermentation are key indicators of gel network development in fermented milk and directly determine the product's fundamental textural characteristics. In this study, the I + P group demonstrated distinct micro-rheological behavior compared with the single-strain groups, characterized by earlier gelation and a denser, more compact gel structure. These results underscore the specific contribution of *B. animalis* subsp. *lactis* IMAU12267 to gel formation, likely associated with bifidobacterial EPS production (Sadeghi et al., 2024). This interpretation is consistent with the observations of Yang et al. (2022), who reported enhanced gel strength and network integrity under co-fermentation conditions. By correlating gelation kinetics derived from microrheological measurements with fermentation time, the present work provides strain-specific evidence that IMAU12267 facilitates the development of the gel network in systems containing commercial starter cultures. The accelerated gelation observed for the I + P group closely paralleled its significantly improved fermentation efficiency. Fermentation was completed in 5.52 h in the I + P group, significantly faster than in either the I or P groups (*P* < 0.05). This finding agrees with Zhong et al. (2025), who showed that co-fermented microbial strains can accelerate acidification and overall fermentation kinetics. The PYS-010 starter likely plays a central role by rapidly lowering pH and modulating redox conditions, establishing an environment conducive to the growth and metabolic activity of *B. animalis* subsp. *lactis*. Similarly, IMAU12267 may enhance the structural stability of the gel matrix through its metabolic activity, improving both fermentation performance and final texture characteristics.

Storage stability is a critical factor influencing both the industrial feasibility and consumer acceptance of fermented milk. Over the 21-day storage period, the I + P group demonstrated significant physicochemical stability and enhanced bacterial viability. By the end of storage, the viable cell count of *B. animalis* subsp. *lactis* IMAU12267 in the I + P group remained at 1.14 × 10^7^ CFU/mL by the end of storage, significantly exceeding that of the single-strain I group (6.76 × 10^6^ CFU/mL) and meeting the national requirement for viable cell counts (≥10^7^ CFU/mL) specified in China's GB19302–2010 standard. This improved survival is likely attributable to metabolic cooperation between strains: LAB in PYS-010 may supply free amino acids that support bifidobacterial growth (Dudriková et al., 2017), while oxygen consumption by *Streptococcus thermophilus* helps create a low-oxygen environment favorable for strictly anaerobic *Bifidobacterium* (Ma, Li, et al., 2025). Throughout storage, the I + P group maintained a stable pH above 4.10 and a TA below 100°T, both within ranges considered optimal for consumer acceptance. In comparison, the I group demonstrated more pronounced post-acidification, which, although contributing to the characteristic tartness of fermented milk, may impair sensory quality when excessive. This observation is consistent with findings reported by Li, Liu, et al. (2025) for a co-fermentation system involving *Lactobacillus helveticus* H11. The I + P group also showed consistently higher WHC and viscosity, both peaking on day 7. Texture profile analysis further demonstrated significantly greater hardness, consistency, and cohesiveness in the I + P group than the I group, indicating that co-fermentation promotes gel network formation and strengthens protein matrix stability through synergistic microbial interactions. Sensory evaluation closely mirrored these physicochemical trends. The I + P group consistently achieved the highest sensory scores, reaching 92.1 on day 7, and displayed a smooth texture, balanced acidity, and a clean, harmonious flavor profile that panelists unanimously favored. These results demonstrate that combining IMAU12267 with PYS-010 effectively moderates acidity fluctuations, alleviates sensory limitations associated with single-strain fermentation, and enhances both textural and flavor attributes. These results support previous reports that co-fermentation improves sensory performance and overall product quality (Li, Liu, et al., 2025) and underscore the potential of IMAU12267 as a valuable strain for the development of premium fermented milk products.

To further elucidate the molecular mechanisms underlying the observed quality differences, untargeted metabolomic profiling was conducted on samples from all three groups collected on day 7, selected as the representative storage point because quality parameters displayed the most distinct intergroup separation at this stage. A total of 32 differential metabolites were identified, and pairwise comparisons of I + P versus I and I + P versus P revealed 14 metabolites with consistent trends, including seven with increased and seven with decreased relative abundances. These findings indicate coordinated metabolic complementarity between *B. animalis* subsp. *lactis* IMAU12267 and PYS-010, which together contribute to the enhanced sensory and physicochemical characteristics of the co-fermented product. Metabolites showing elevated levels appear to play direct roles in improving flavor, nutritional value, and functional properties in the I + P group. For example, 2-pentanone, a volatile ketone with a low odor threshold, contributes to the sweet aroma typical of high-quality dairy products and can be produced by fatty acid β-oxidation, amino acid degradation, or microbial metabolism (Li et al., 2022). Phenyllactic acid, another upregulated compound produced by lactic acid bacteria via lactate dehydrogenase, shows milder acidity than acetic or lactic acid and can both moderate post-acidification and inhibit spoilage microorganisms, extending shelf life (Zhou et al., 2025). Increased concentrations of α-lactose and D-galactose in the I + P group likely enhance both the flavor and nutritional attributes. *Streptococcus thermophilus* and *Bifidobacterium* species can hydrolyze lactose into glucose and galactose through β-galactosidase activity (Park et al., 2019). Galactose functions as an essential carbon source for microbial growth and as a precursor for EPS biosynthesi. Although EPS content was not measured directly, previous studies indicate that *B. animalis* subsp. *lactis* possesses a complete EPS biosynthetic gene cluster, and high-EPS-producing strains form denser gel networks, reduce surface hydrophobicity and free thiol levels, and significantly improve WHC and viscosity (Lee & O'Sullivan, 2010; Zhang et al., 2024). Elevated riboflavin (vitamin B₂) concentrations in the I + P group further demonstrate the nutritional enhancement achieved through co-fermentation. Riboflavin is vital for human health and has been associated with reduced risk of cancer, cardiovascular disease, and visual disorders (Li, Liu, et al., 2025). Its increased abundance likely reflects synergistic conversion of precursor compounds during co-fermentation by IMAU12267 and PYS-010. Similarly, higher levels of uridine 5′-monophosphate and capric acid may contribute to improved functional properties. Uridine 5′-monophosphate supports intestinal health (Li et al., 2019), whereas capric acid, a medium-chain fatty acid, serves as a rapidly used energy substrate via mitochondrial β-oxidation (Schonfeld & Wojtczak, 2016). This efficient energy supply may facilitate faster growth and metabolic activity of IMAU12267 in the co-fermentation system, enhancing fermentation efficiency and sustaining high viable cell counts.

Metabolites with reduced relative abundance appear to play protective roles in preserving product quality and maintaining flavor balance. In particular, the moderated decrease in acetic acid is critical for sensory stability. In *B. animalis* subsp. *lactis*, acetic acid is mainly generated via the Bifid shunt pathway, in which fructose-6-phosphate phosphoketolase (F6PPK) converts carbohydrates such as lactose into acetic and lactic acids at a theoretical molar ratio of 3:2 (Van der Meulen et al., 2006). In the single-strain I group, the absence of metabolic modulation by PYS-010 likely permits excessive acetic acid accumulation, promoting accelerated post-acidification, disruption of the gel network, reduced WHC, and the emergence of off-flavors. High acetic acid levels have been reported to dominate volatile profiles, masking desirable aroma compounds such as acetaldehyde and weakening the characteristic flavor of fermented milk (Fontes et al., 2023), which explains the comparatively lower sensory scores observed for the I group during storage. In comparison, the I + P group maintained acetic acid within an optimal range, likely because PYS-010 channels carbon metabolism predominantly through the Embden–Meyerhof–Parnas (EMP) pathway to generate lactic acid, redirecting carbon flux away from acetic acid production (Zhang et al., 2016). This metabolic regulation helps prevent flavor imbalance, stabilizes post-acidification, and allows acetic acid to contribute harmoniously to the overall flavor profile. Notably, given its relatively high pKa, acetic acid may also retain comparatively stronger antimicrobial activity under low-pH conditions (Guimarães et al., 2018). Thus, beyond its sensory role, it may also enhance shelf-life stability in the I + P group. Further improvements in aroma quality may be linked to reduced levels of 1H-indole-3-carboxaldehyde and 5-methoxyindoleacetate, both products of tryptophan metabolism whose declines likely limit the formation of indole-related off-odors (Ferrer et al., 2022; Liu et al., 2020; Ma, Zhao, et al., 2025). The observed decrease in 3′-sialyllactose, a major bovine milk oligosaccharide (BMO), suggests more efficient utilization of this compound as a carbon source during co-fermentation, reflecting effective cross-feeding interactions between IMAU12267 and PYS-010 (Bondue et al., 2016). Variation in the dipeptide cysteinyl-asparagine may be associated with proteolytic activity (Caminero et al., 2023), and its reduced abundance in the I + P group could indicate consumption as a growth factor by *Streptococcus thermophilus* within PYS-010, consistent with observations reported by Li, Liu, et al. (2025). These metabolomic results provide compelling molecular evidence that co-fermentation of IMAU12267 with PYS-010 establishes a synergistic metabolic network. Such cooperation enhances nutrient biosynthesis, optimizes organic acid balance, and suppresses undesirable oxidative pathways, ultimately improving the flavor, texture, and nutritional value of the fermented milk.

From a metabolic pathway integration standpoint, the observed differences in metabolite abundances did not occur in isolation. Instead, they represented coordinated metabolic patterns that corresponded with the physicochemical and sensory advantages displayed by the I + P group on day 7 of storage. For example, α-lactose and D-galactose were mapped to galactose metabolism, and their elevated levels were consistent with the higher WHC and viscosity observed in the I + P group, suggesting that shifts in carbohydrate metabolism may be linked to the structural properties of the gel network. Uridine 5′-monophosphate, riboflavin, and capric acid were associated with pyrimidine metabolism, riboflavin metabolism, and fatty acid biosynthesis, respectively; their increased abundances provide metabolomic evidence supporting enhancements in nutritional and functional attributes in the co-fermented samples. In comparison, acetic acid and the indole-derived metabolites 1H-indole-3-carboxaldehyde and 5-methoxyindoleacetate were assigned to glycolysis/gluconeogenesis and tryptophan metabolism pathways, and their reduced levels aligned with the superior sensory scores of the I + P group. This pattern suggests that co-fermentation may promote a more balanced flavor profile, potentially by regulating the synthesis and accumulation of organic acids and nitrogen-containing compounds. Pathway annotations and the coordinated variation of these core metabolites indicate metabolic complementarity and synergistic interactions between IMAU12267 and PYS-010 within the co-fermentation system. Although these findings provide initial evidence of the metabolic advantages of co-fermentation, several limitations should be noted. The present validation was conducted using a single strain combination under fixed processing conditions; therefore, its general applicability to other strain pairings or production parameters remains to be determined. Moreover, the key metabolites identified through untargeted metabolomics require further confirmation by targeted quantification and mechanistic investigation. Future studies should expand the diversity of strain combinations and processing conditions, incorporate multi-omics approaches to quantitatively assess critical metabolites, and employ in vivo models to assess nutritional and health effects systematically. Such efforts would enable a more comprehensive understanding of IMAU12267's functional potential and facilitate its application in the development of advanced fermented milk products.

## 5 Conclusions

This study systematically investigated the impact of co-fermentation with *Bifidobacterium animalis* subsp. *lactis* IMAU12267 and the commercial starter culture PYS-010 on the physicochemical, textural, sensory, and metabolic properties of fermented milk. The findings showed that incorporating IMAU12267 significantly enhanced fermentation efficiency, significantly shortening fermentation time in the I + P group. Simultaneously, co-fermentation also improved gelation performance and reinforced the structural integrity of the final product. Throughout 21 days of storage, the I + P group maintained higher viable cell counts of IMAU12267 and demonstrated reduced post-acidification, resulting in improved WHC, viscosity, texture, and sensory quality. Metabolomic analysis at day 7 identified seven key functional metabolites associated with desirable product attributes, including enhanced flavor (2-pentanone, phenyllactic acid), improved texture (α-lactose, D-galactose), and increased nutritional value (riboflavin, uridine 5′-monophosphate, capric acid). Co-fermentation suppressed the accumulation of unfavorable flavor-related compounds, such as acetic acid, 2-hexenoylcarnitine, 9-oxooctadecanoic acid, 1H-indole-3-carboxaldehyde, and 5-methoxyindoleacetate. These results indicate that IMAU12267 functions as an effective adjunct starter, enhancing both technological performance and functional quality in fermented milk. Its synergistic interaction with PYS-010 provides a robust theoretical basis and practical strategy for the development of next-generation fermented dairy products with superior quality and added functionality.

The following are the supplementary data related to this article.

Supplementary Fig. S1 Fermentation characteristics and metabolomics data quality assessment of fermented milk. (A) Fermentation time of fermented milk prepared using a commercially available starter PYS-010 (P), IMAU12267 (I), or their combination (I+P). Different uppercase letters represent significant differences within groups (*P* < 0.05). (B) Total ion current chromatogram in negative ion mode (NEG). (C) Total ion current chromatogram in positive ion mode (POS). (D) Cross-validation plot of the PLS-DA model. (E) Permutation test results for model validation. Supplementary Fig. S1

## CRediT authorship contribution statement

**Xiao Liu:** Writing – review & editing, Writing – original draft, Software, Conceptualization. **Sheng Zhang:** Visualization, Investigation. **Lu Li:** Software, Methodology. **Yongfu Chen:** Validation, Funding acquisition. **Jianli Li:** Supervision, Funding acquisition.

## Declaration of competing interest

The authors declare that they have no known competing financial interests or personal relationships that could have appeared to influence the work reported in this paper.

## Data Availability

Data will be made available on request.
