# Supplementary figures and images for: Effects of Bifidobacterium animalis subsp. lactis IMAU12267 on milk fermentation, subsequent storage, and functional properties
Source: Food Chem X. 2026 Mar 26;35:103791. doi: 10.1016/j.fochx.2026.103791 (PMC13068531; doi:10.1016/j.fochx.2026.103791)

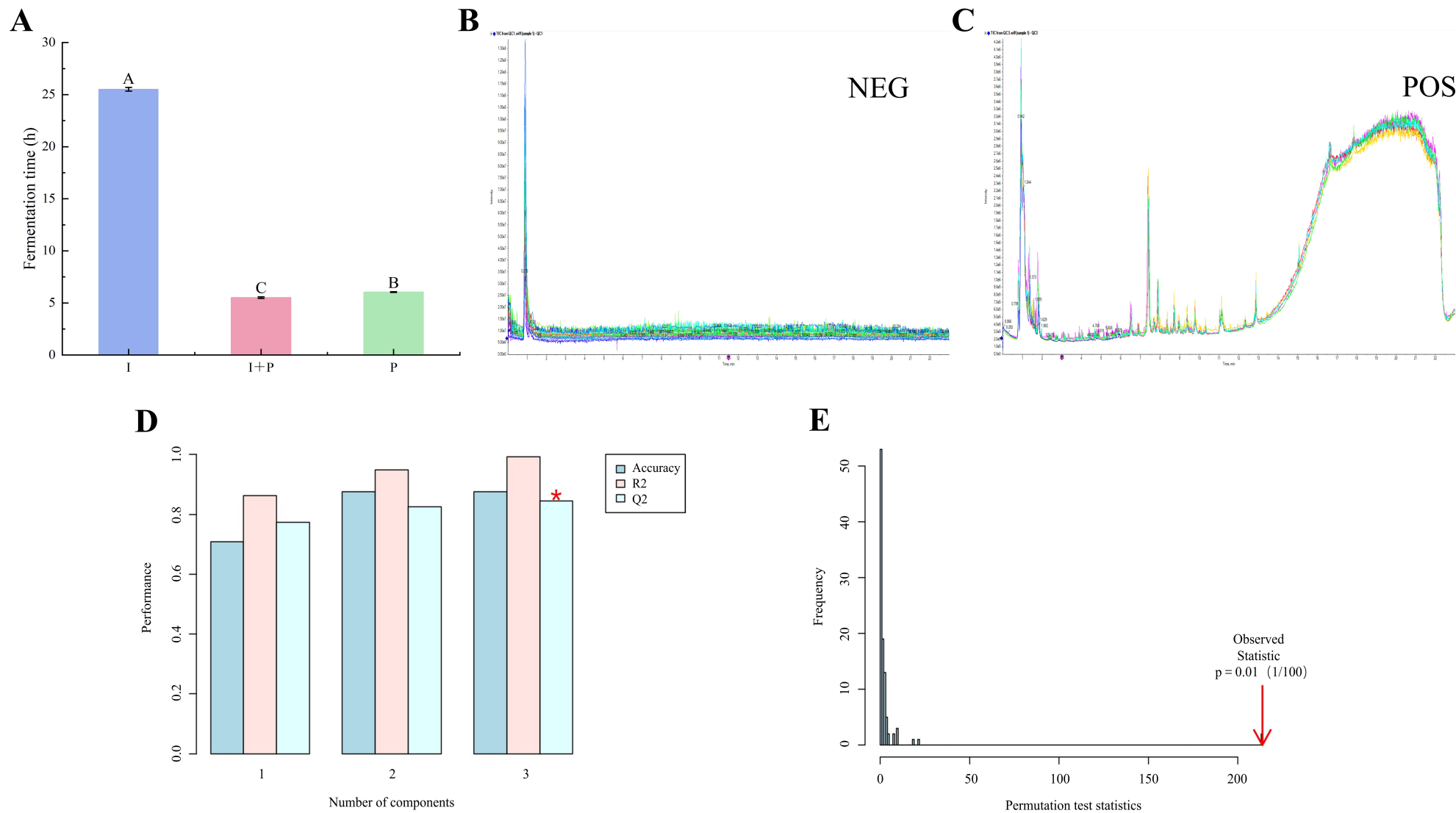

Supplement: Supplementary Fig. S1 — Fermentation characteristics and metabolomics data quality assessment of fermented milk. (A) Fermentation time of fermented milk prepared using a commercially available starter PYS-010 (P), IMAU12267 (I), or their combination (I+P). Different uppercase letters represent significant differences within groups (P < 0.05). (B) Total ion current chromatogram in negative ion mode (NEG). (C) Total ion current chromatogram in positive ion mode (POS). (D) Cross-validation plot of the PLS-DA model. (E) Permutation test results for model validation. [file mmc1.pdf]
